# Brain natriuretic peptide as a biomarker for predicting contrast-induced nephropathy in patients undergoing coronary angiography/intervention: A systematic review and meta-analysis

**DOI:** 10.1097/MD.0000000000032432

**Published:** 2022-12-30

**Authors:** Xuefeng Wu, Xili Yang, Zhaoyan Xu, Jianming Li

**Affiliations:** a Department of Cardiology, First People’s Hospital of Foshan, Foshan City, Guangdong, China.

**Keywords:** brain natriuretic peptide, contrast-induced nephropathy, meta-analysis

## Abstract

Contrast-induced nephropathy (CIN) is associated with adverse events. As there are no effective treatments, the early identification of high-risk patients is required. Individual studies have suggested the utility of brain natriuretic peptide in predicting CIN. Therefore, this meta-analysis aimed to systematically investigate the value of brain natriuretic peptide in predicting CIN in patients undergoing coronary angiography (CAG) or percutaneous coronary intervention (PCI).

**Methods:**

We searched PubMed, Embase, Cochrane Central Register of Controlled Trials Library, and Web of Science from inception date to March 9, 2022. Studies that evaluated the predictive value of brain natriuretic peptide for CIN outcomes in patients after CAG or PCI were included. The quality of the included studies was assessed using the QUADAS-2 tool. Diagnostic accuracy estimates were calculated using a random-effects model. Subgroup and meta-regression analyses were performed to identify the potential sources of heterogeneity.

**Results:**

Twelve studies with 7789 patients were included in the meta-analysis. The pooled sensitivity and specificity of brain natriuretic peptide for the prediction of CIN were 0.73 (95% CI: 0.67–0.78) and 0.77 (95% CI: 0.71–0.82), respectively. The area under the summary receiver operating characteristic curve was 0.80 (95% CI: 0.77–0.84). Meta-regression analysis indicated that the sources of sensitivity heterogeneity may be the country, mean age, and study population. Additionally, country, study population, study design, and index text contributed to the specificity heterogeneity.

**Conclusion:**

This study demonstrated that brain natriuretic peptide could function as a novel potential marker for the early detection of CIN in patients undergoing CAG or PCI.

## 1. Introduction

According to the Global Burden of Disease Study (2019), coronary artery disease (CAD) is the leading cause of deaths worldwide.^[1]^ As the gold standard, coronary angiography (CAG) plays a key role in the diagnosis of CAD, and percutaneous coronary intervention (PCI) is one of the most important treatments for CAD recommended by the guidelines.^[2,3]^ However, patients may develop contrast-induced nephropathy (CIN) after CAG or PCI, which is the third most common cause of hospital-acquired acute kidney injuries,^[4]^ which prolongs hospital stay duration and significantly correlates with increased mortality, myocardial infarction, as well as stent thrombosis.^[5,6]^ Since CIN does not have effective therapies, the early identification of patients at high risk of CIN occurrence is extremely important. A variety of risk factors for CIN have been identified, such as impaired renal function, diabetes mellitus, age, heart failure, and effective intravascular volume depletion.^[7]^ Many risk-predictive models have been developed to stratify the risk of CIN^[8–10]^; however, certain variables included in these models, such as the volume of contrast, can only be obtained post-procedure, limiting their usefulness as guidance for preventing CIN before CAG or PCI. In the past few years, significant progress has been made in the field of biomarkers for early detection of CIN. Brain natriuretic peptide, a biomarker for cardiac function, is widely used in clinical practice and is closely related to the outcomes of patients with CAD.^[11,12]^ It reflects the state of ventricular wall tension and volume load, and is associated with the renin-angiotensin and sympathetic nervous systems which contribute to the development of CIN.^[7]^ Studies have reported that the brain natriuretic peptide can be viewed as a predictor of CIN. A systematic review and meta-analysis was recently conducted to assess the diagnostic value of brain natriuretic peptide for CIN^[13]^; however, the meta-analysis focused only on patients with acute coronary syndrome patients, which refers to a small proportion of patients undergoing interventional therapy. Evidence that brain natriuretic peptide predicts CIN in a wider range of patients is limited. Therefore, we performed this review to evaluate the CIN predictive capacity of brain natriuretic peptide in patients undergoing CAG or PCI.

## 2. Methods

This systematic review and meta-analysis were conducted according to the Preferred Reporting Items for Systematic reviews and meta-analysis (PRISMA) guidelines.^[14]^ The protocol for this study was registered under PROSPERO registration number CRD42022318275. Ethical approval was not required because our study was based on published data and individual patient data were not included.

## 3. Search strategy

We searched PubMed, Embase, Cochrane Central Register of Controlled Trials Library, and Web of Science from the inception date to March 9, 2022. The search strategy was based on Mesh terms and keywords from titles and abstracts without language restrictions. Additionally, we manually screened the references of the included studies for potential studies. The search strategy and exact results for PubMed are shown in Table 1.

**Table 1:** The search terms and exact results in PubMed database.

Database	#	Search strategy	Results
PubMed	1	“natriuretic peptide, brain”[MeSH Terms]	15,708
	2	“brain”[Title/Abstract] OR “pro brain”[Title/Abstract] OR “pro b type”[Title/Abstract] OR “b type”[Title/Abstract] OR “type b”[Title/Abstract] OR “ventricular”[Title/Abstract]	1,487,342
	3	“natriuretic peptide*”[Title/Abstract]	33,584
	4	#2 and #3	22,403
	5	“nt bnp”[Title/Abstract] OR “n bnp”[Title/Abstract] OR “ntprobnp”[Title/Abstract] OR “nt probnp”[Title/Abstract] OR “pro bnp*”[Title/Abstract] OR “probnp*”[Title/Abstract] OR “bnp”[Title/Abstract]	18,739
	6	“natriuretic factor”[Title/Abstract]	4,464
	7	#1 or #4 or #5 or #6	34,067
	8	“kidney diseases”[MeSH Terms]	548,322
	9	“nephropathy”[Title/Abstract] OR “kidney diseases”[Title/Abstract] OR “kidney disease”[Title/Abstract]	153,768
	10	“acute kidney injury”[MeSH Terms]	51,927
	11	“renal diseases”[Title/Abstract] OR “renal disease”[Title/Abstract] OR “acute renal failure”[Title/Abstract] OR “acute kidney failure”[Title/Abstract] OR “acute kidney insufficiency”[Title/Abstract] OR “acute renal insufficiency”[Title/Abstract] OR “acute renal injury”[Title/Abstract] OR “acute kidney injury”[Title/Abstract]	126,171
	12	#8 OR #9 OR #10 OR #11	630,424
	13	“contrast media”[MeSH Terms]	93,039
	14	“radiopaque”[Title/Abstract] OR “radiocontrast”[Title/Abstract] OR “contrast agent”[Title/Abstract] OR “contrast material”[Title/Abstract] OR “contrast medium”[Title/Abstract] OR “contrast media”[Title/Abstract] OR “contrast”[Title/Abstract]	1,083,897
	15	#13 OR #14	111,632
	16	#12 AND #15	26,903
	17	“CI AKI”[Title/Abstract] OR “CIN”[Title/Abstract]	11,924
	18	#16 OR #17	36,910
	19	#7 AND #18	136

## 4. Inclusion and exclusion criteria

Studies were selected based on the following inclusion criteria: the ability of natriuretic peptide to predict CIN occurrence among patients receiving CAG or PCI was reported; a 2 × 2 table containing true positive, true negative, false positive, and false negative data was constructed; and CIN was clearly defined. Systematic reviews, case reports, comments, letters, conference abstracts, and animal studies were excluded. Two authors (Wu and Xu) independently assessed the included studies. Any disagreements were resolved by discussion or consultation with a third author (Yang) if necessary.

## 5. Data extraction

Two authors (Wu and Xu) independently conducted data extraction and discrepancies were resolved by consensus. For each study, we extracted the following information: 1st author, year of publication, study design, country, study population, sample size, sex, average age, definition of CIN, incidence of CIN, index test, cutoff point, area under the receiver operating characteristic curve (AUC), sensitivity, specificity, true positive, true negative, false positive, and false negative.

## 6. Quality assessment

Two authors (Wu and Xu) independently assessed the quality of the included studies using the QUADAS-2 tool. Any disagreement was resolved by consultation with a third author (Yang). The QUADAS-2 tool comprises 4 key domains: patient selection, index test, reference standard, flow, and timing.^[15]^

## 7. Statistical analysis

The Spearman rank correlation coefficient was calculated to evaluate the threshold effects. A threshold effect was considered to exist if the *P* value was < 0.05. A random-effects model was used to calculate pooled diagnostic measures, including sensitivity, specificity, positive likelihood ratio, negative likelihood ratio, and diagnostic odds ratio. A summary receiver operating characteristic curve was constructed to estimate overall diagnostic accuracy. We used the inconsistency (*I*^*2*^) test to assess the heterogeneity between studies. An *I*^*2*^ value of > 50% indicated substantial heterogeneity. Meta-regression and subgroup analyses were performed to determine sources of heterogeneity. The variables included in the regression analysis were the study design, country, study population, index test, sample size, and average age. Sensitivity analysis was conducted to detect the impact of each study on the meta-analysis results. Publication bias was assessed using the Deek’s funnel plots. All data were analyzed using STATA (version 16.0), Review Manager 5.4, and MetaDisc 1.4.

## 8. Results

### 8.1. Search results and study characteristics

**Figure 1.**
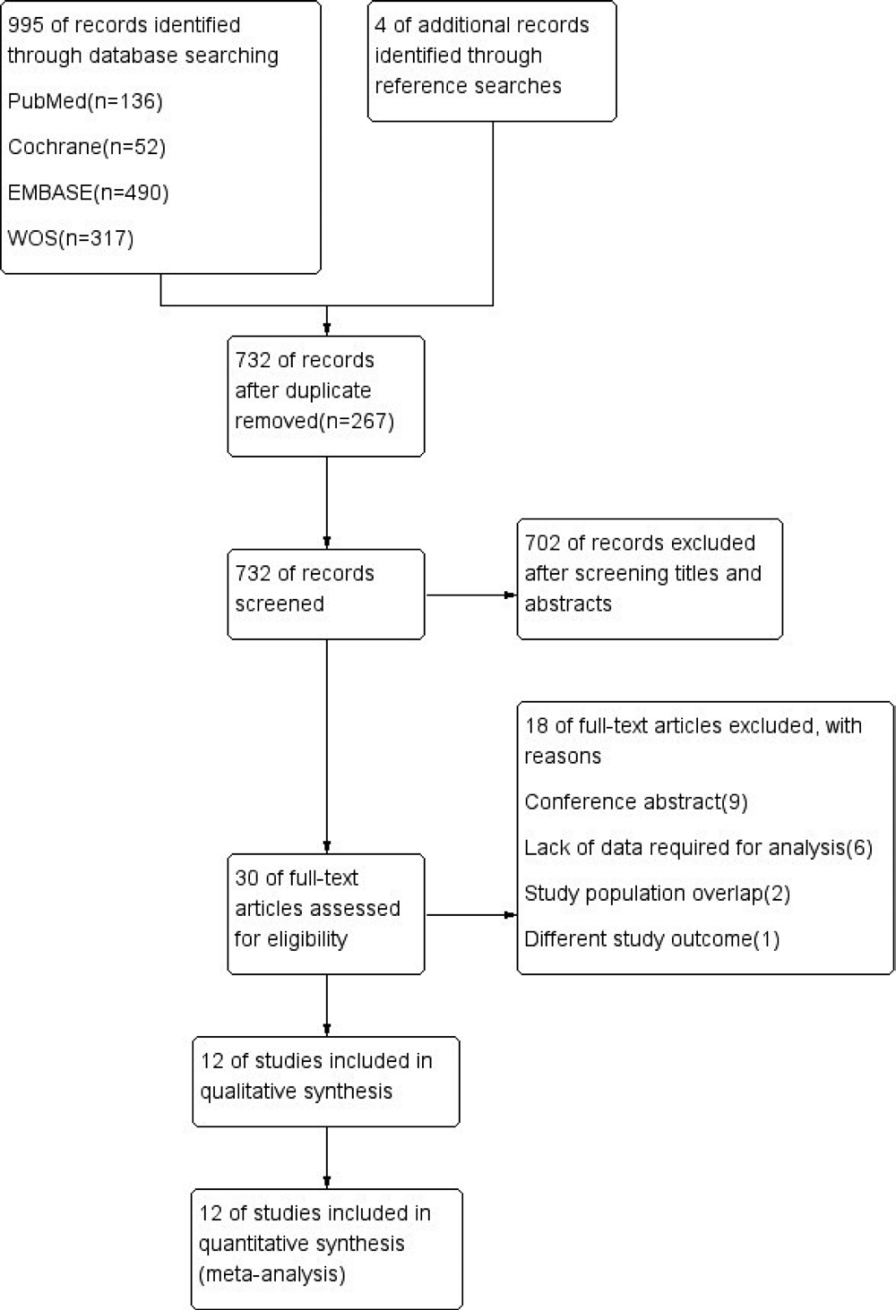
PRISMA flow diagram of the study selection process.

A total of 999 records were identified through database and reference searches, and 267 duplicate studies were removed. After screening titles and abstracts, 702 records were excluded. The remaining 30 studies were included in the full-text evaluation. Nine conference abstracts were excluded from the analysis. Six studies were excluded due to the lack of data required for analysis. Two studies were excluded because of participant overlap. One study was excluded because it used a different definition of kidney injury, which varied greatly in clinical practice. As a result, 12 studies with 7789 patients were included in this meta-analysis (Fig. 1).

The basic characteristics of the included studies are presented in Table 2 to 3. All studies were published between 2014 and 2021. Nine studies were prospectively designed, and the remaining 3 were retrospective. Seven studies enrolled patients with ST-segment elevated myocardial infarction (STEMI) or non-ST-segment elevated myocardial infarction. The incidence of CIN ranged from 1.2% to 19.6%. Eight studies used N-terminal pro-B-type natriuretic peptide (NT-proBNP) and 4 used B-type natriuretic peptide (BNP) as an index test. The cutoff values reported in these studies varied from 42.4 to 676 pg/mL for BNP and from 512 to 2320 pg/mL for NT-proBNP.

**Table 2:** Characteristics of included studies.

Study	Study design	Population	Country	Total cases	Male	Mean age	Definition of CIN	CIN incidence
Agarwal 2018^[16]^	prospective	STEMI/NSTEMI underwent PCI	India	150	97	63.0 ± 9.07	An absolute increase of ≥ 0.5 mg/dL or a relative increase of ≥ 25% within 48 h after index angiography	14.60%
Akgul 2014^[17]^	prospective	STEMI underwent PCI	Turkey	424	340	55.3 ± 12.0	An absolute increase of ≥ 0.3 mg/dL or a relative increase of ≥ 50% from the baseline	13.70%
Alan 2019^[18]^	Prospective	ACS underwent PCI	France	216	172	62.7 ± 12.3	An absolute increase of ≥ 0.3 mg/dL or a relative increase of ≥ 50% within 7 d after injection	10%
Kurtul 2014^[19]^	Prospective	STEMI/NSTEMI underwent PCI	Turkey	436	280	62.27 ± 13.01	An increase of ≥ 0.5 mg/dL or ≥ 25% above baseline within 72 h after contrast administration	14.40%
Lin 2018^[20]^	Prospective	CAD underwent PCI	China	540	386	78.86 ± 3.29	An absolute increase of ≥ 0.3 mg/dL or a relative increase of ≥ 50% within 48 h after contrast medium exposure	10%
Liu 2015^[21]^	Prospective	Patients underwent CAG or PCI	China	2248	1677	63.5 ± 10.7	An increase of > 0.5 mg/dL over the baseline value within 48–72 h after the administration of contrast medium	2.20%
Liu 2016^[22]^	Prospective	STEMI underwent PCI	China	283	NA	62.9 ± 12.3	An absolute increase of ≥ 0.5 mg/dL from baseline within 48–72h after contrast medium exposure	9.20%
Mo 2021^[23]^	Retrospective	CAD underwent CAG or PCI	China	2009	1588	63.3 ± 9.9	An increase of 0.3 mg/dL or ≥ 50% at 48 h after surgery	1.20%
Moltrasio 2014^[24]^	Prospective	STEMI/NSTEMI underwent PCI	Italy	639	484	65.5 ± 13.1	An increase of ≥ 0.3 mg/dL during CCU stay from baseline	13%
Parenica 2020^[25]^	Retrospective	STEMI underwent PCI	Czech	427	328	(–)	An increase of ≥ 0.3 mg/dL or ≥ 50% from baseline	8.90%
Tung 2015^[26]^	Prospective	STEMI underwent PCI	China	189	163	62.6 ± 13.9	An increase of ≥ 0.3 mg/dl or ≥ 50% from baseline within 48 hours	19.60%
Lu 2018^[27]^	Retrospective	Patients with chest pain underwent CAG	China	228	136	63.8 ± 9.70	An absolute increase of ≥ 0.5 mg/dL or a relative increase of ≥ 25% within 72 h after index angiography	15.79%

ACS = acute coronary syndrome, CAD = coronary artery disease, CAG = coronary artery angiography, CIN = contrast induced nephropathy, NSTEMI = non ST-segment elevated myocardial infarction, PCI = percutaneous coronary intervention, STEMI = ST-segment elevated myocardial infarction.

**Table 3:** The detailed information about index test of included studies.

Study	Index test	Cut off (pg/mL)	AUC	SEN	SPE	TP	FP	TN	FN
Agarwal 2018^[16]^	NT-proBNP	2320	0.917	90.9	81.5	20	24	104	2
Akgul 2014^[17]^	BNP	42.4	0.65	60	61	35	143	223	23
Alan 2019^[18]^	NT-proBNP	512	0.79	81	66	17	66	129	4
Kurtul 2014^[19]^	NT-proBNP	2149	0.828	79.4	74.3	50	96	277	13
Lin 2018^[20]^	NT-proBNP	1133	0.719	66.7	70.8	36	142	344	18
Liu 2015^[21]^	NT-proBNP	682	0.766	78	70	39	659	1539	11
Liu 2016^[22]^	NT-proBNP	1800	0.76	69	70	18	77	180	8
Mo 2021^[23]^	NT-proBNP	847	NA	56.5	89.5	14	208	1777	10
Moltrasio 2014^[24]^	BNP	184	0.702	79	74	67	144	410	18
Parenica 2020^[25]^	BNP	623	0.75	57.9	88.2	22	46	343	16
Tung 2015^[26]^	BNP	676	0.86	0.75	0.89	27	17	136	9
Lu 2018^[27]^	NT-proBNP	(–)	0.821	77.8	78	28	42	150	8

AUC = area under curve, BNP = b-type natriuretic peptide, FN = false negatives, FP = false positives, NT-proBNP = N-terminal pro-B-type natriuretic peptide, SEN = sensitivity, SPE = specificity, TN = true negatives, TP = true positives.

### 8.2. Quality evaluation

After careful evaluation of the methodological quality of the included studies, we found 1 study had a high risk of bias for patient selection. This is because the study only enrolled patients eligible for optical coherence tomography angiography. Three studies had an unclear risk of bias in patient selection. Overall, most of the literature had a low risk of bias and applicability concerns (Figure S1, Supplemental Digital Content, http://links.lww.com/MD/I188).

### 8.3. Threshold and diagnostic accuracy of brain natriuretic peptide for the prediction of CIN

**Figure 2.**
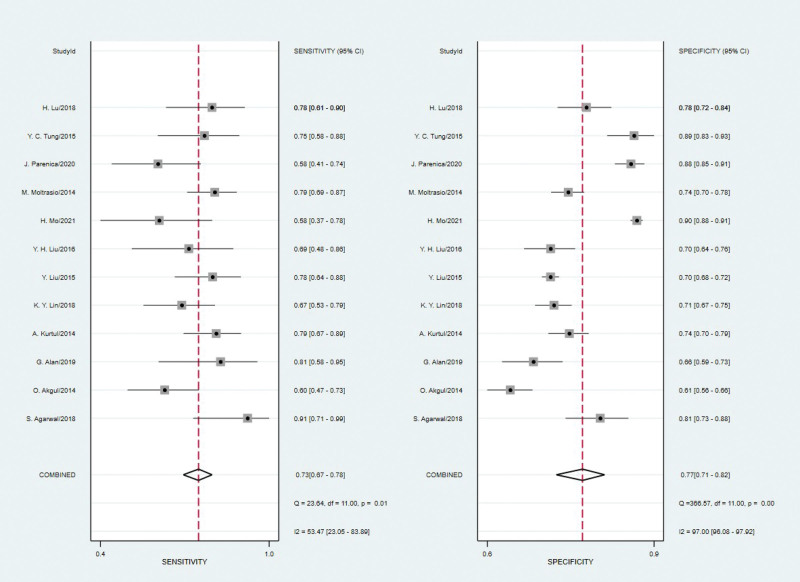
Forest plot of the sensitivity and specificity of brain natriuretic peptide for the prediction of CIN. CIN = contrast-induced nephropathy.

**Figure 3.**
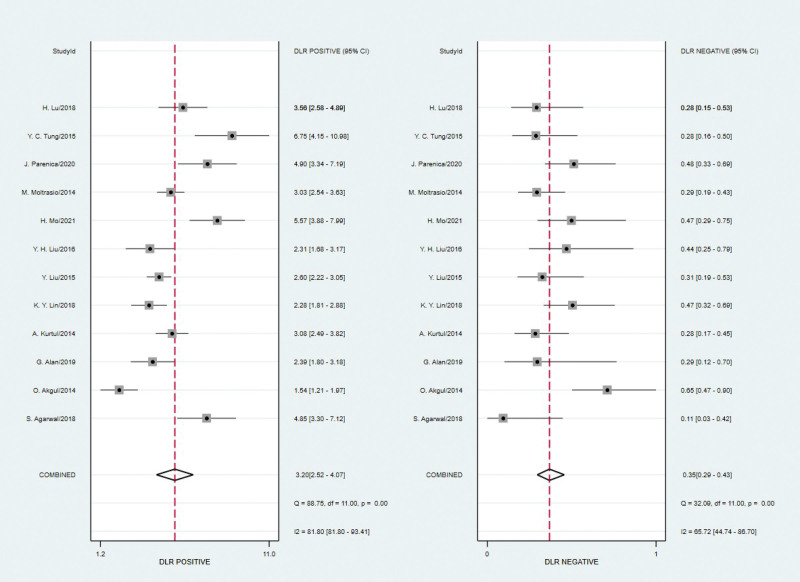
Forest plot of the positive likelihood ratio and negative likelihood ratio of brain natriuretic peptide for predicting CIN. CIN = contrast-induced nephropathy.

**Figure 4.**
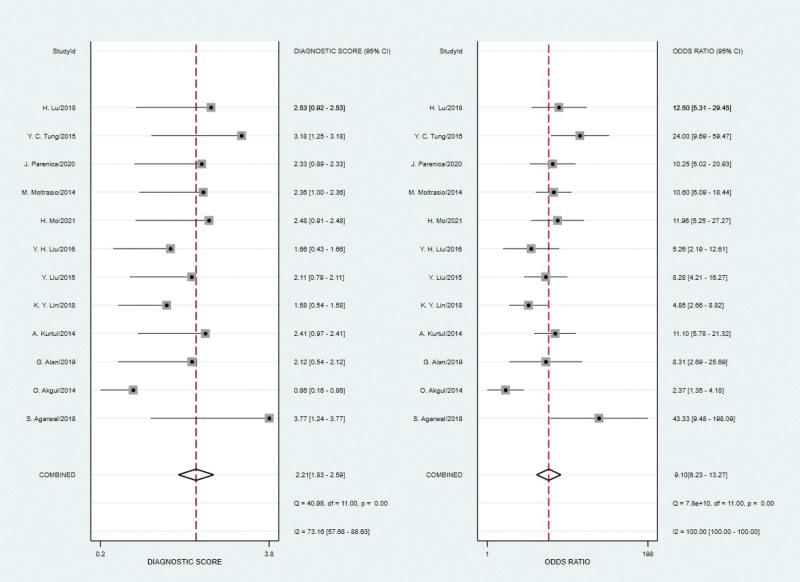
Forest plot of the diagnostic score and diagnostic odds ratio (DOR) of brain natriuretic peptide for the prediction of CIN. CIN = contrast-induced nephropathy, DOR = diagnostic odds ratio.

**Figure 5.**
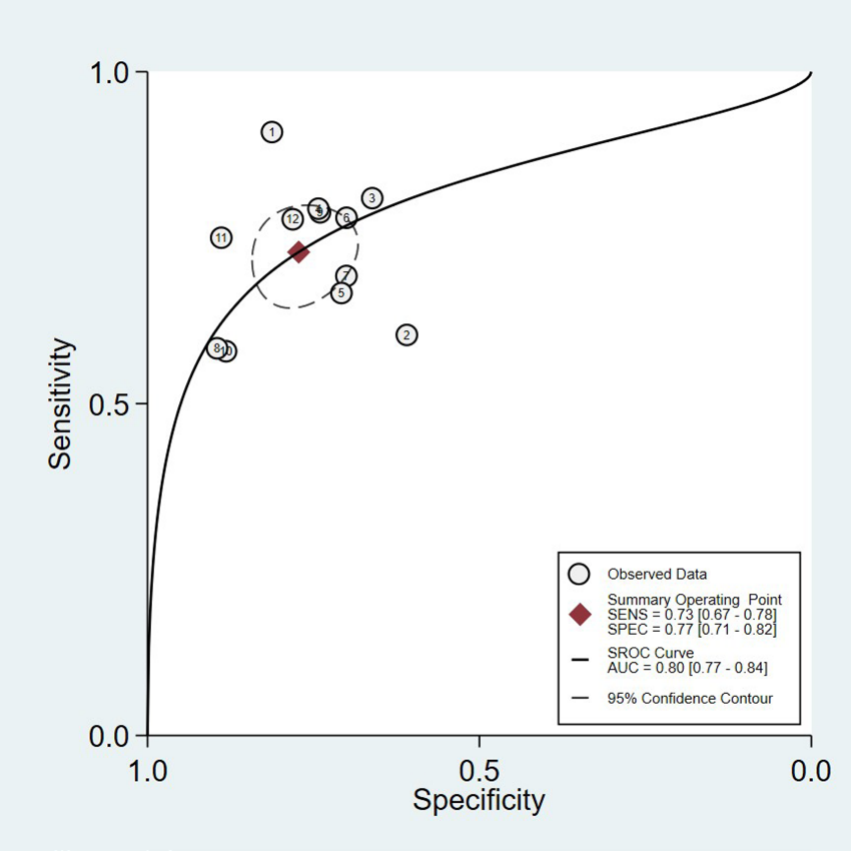
Summary receiver operating curve (SROC) of brain natriuretic peptide for predicting CIN. The area under the receiver operating curve (AUC) was 0.80 (95% CI: 0.77–0.84), indicating that brain natriuretic peptide could predict CIN. AUC = area under receiver operating characteristic curve, CIN = contrast-induced nephropathy, SROC = summary receiver operating characteristic.

Spearman’s correlation coefficient for brain natriuretic peptide was 0.21 with a *P* value of 0.513, indicating that a threshold effect did not exist. As can be seen from the forest plot (Figures 2–4), the combined estimates of brain natriuretic peptide for the prediction of CIN were as follows: sensitivity, 0.73 (95% CI: 0.67–0.78); specificity, 0.77 (95% CI: 0.71–0.82); positive likelihood ratio, 3.2 (95% CI: 2.52–4.07); negative likelihood ratio, 0.35 (95% CI: 0.29–0.43); diagnostic score, 2.21(95% CI: 1.83–2.59); diagnostic odds ratio, 9.10 (95% CI: 6.23–13.27). Summary receiver operating characteristic was drawn to evaluate the accuracy of the brain natriuretic peptide, and the AUC was 0.80 (95% CI: 0.77–0.84) (Fig. 5).

### 8.4. Meta-regression and subgroup analysis

**Figure 6.**
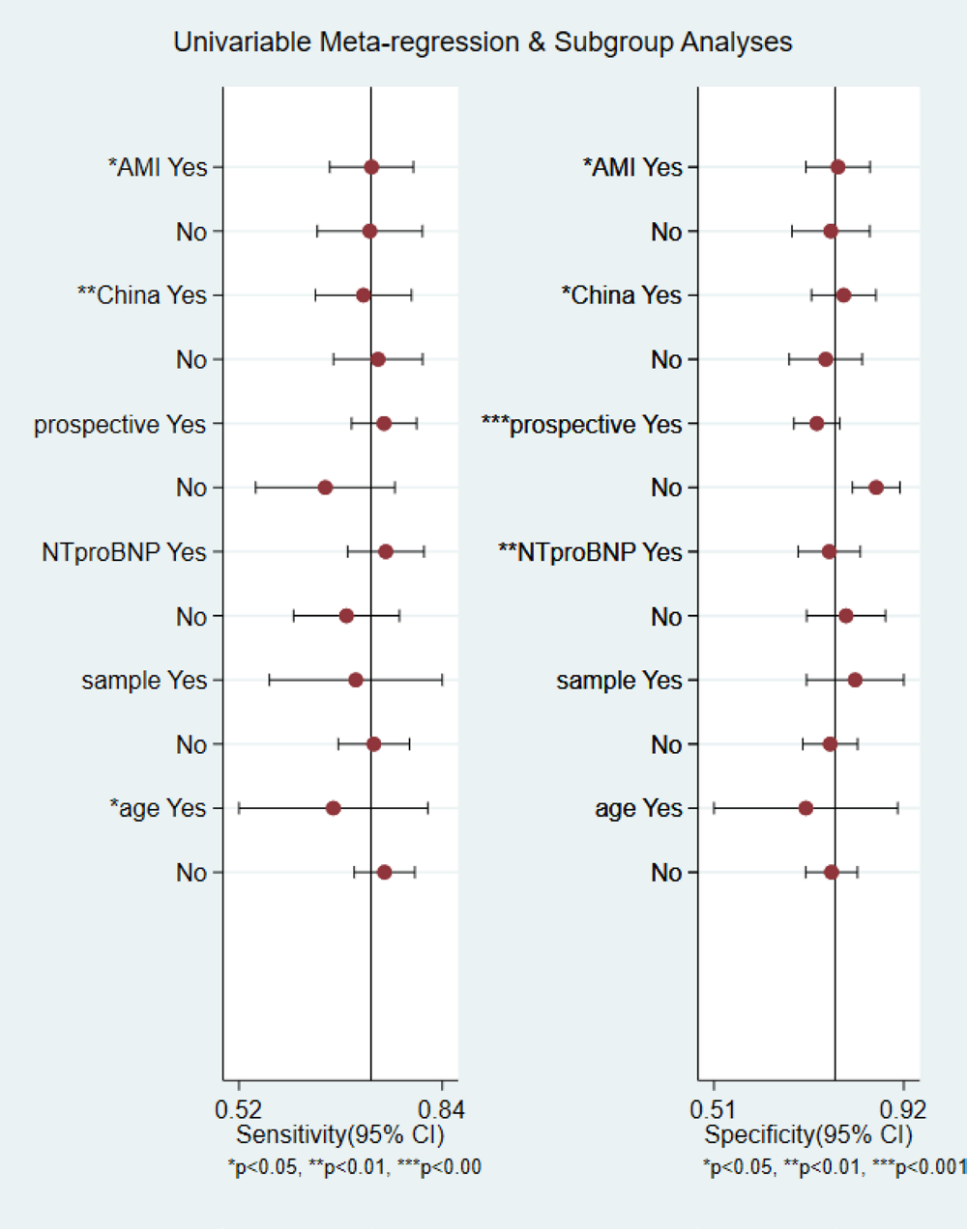
Regression analysis for the prediction of CIN by brain natriuretic peptide. CIN = contrast-induced nephropathy.

Since significant heterogeneity was observed in the sensitivity (*I*^2^ = 53.47%), specificity (*I*^2^ = 97.00%), positive likelihood ratio (*I*^2^ = 81.80%), negative likelihood ratio (*I*^2^ = 65.72%), diagnostic score (*I*^2^ = 73.16%), and diagnostic odds ratio (*I*^2^ = 100.00%), univariate meta-regression and subgroup analyses were conducted to identify potential sources of heterogeneity. The variables included in the regression analysis were study design, country, study population, index test, sample size larger than 2000, and average age over 70 years. Seven studies that enrolled patients with STEMI or non-ST-segment elevated myocardial infarction were classified as acute myocardial infarction group. One study did not report the mean age of the population and was therefore not included in the regression analyses for this characteristic. Univariate regression revealed that country, mean age, and study population may be the main factors of heterogeneity for sensitivity. Additionally, country, study population, study design, and index text contributed to the specificity heterogeneity (Fig. 6). In the subgroup analyses of patient with average age < 70 years, the sensitivity, specificity, and diagnostic odds ratio in predicting CIN were 0.74 (0.67–0.79), 0.78 (0.71–0.83), and 10 (7–14), respectively. In the study design subgroup, the sensitivity and specificity were 0.75 (0.70–0.80) and 0.73 (0.68–0.78) in prospective studies. When NT-proBNP was used, the pooled results indicated sensitivity and specificity were 0.75 (0.69–0.81) and 0.76 (0.69–0.83) (Table 4).

**Table 4:** Subgroup analysis for the prediction of CIN by brain natriuretic peptide.

Subgroup	Number of studies	Sen (95% CI)	Spe (95% CI)	PLR (95% CI)	NLR (95% CI)	DOR (95% CI)
All studies	12	0.73 (0.67–0.78)	0.77 (0.71–0.82)	3.2 (2.5–4.1)	0.35 (0.29–0.43)	9 (6–13)
Prospective	9	0.75 (0.70–0.80)	0.73 (0.68–0.78)	2.8 (2.2–3.5)	0.34 (0.26–0.44)	8 (5–14)
Average age < 70 years	10	0.74 (0.67–0.79)	0.78 (0.71–0.83)	3.3 (2.6–4.3)	0.34 (0.27–0.42)	10 (7–14)
**Country**						
China	6	0.72 (0.64–0.79)	0.79 (0.72–0.86)	3.4 (2.4–4.8)	0.36 (0.29–0.45)	9 (6–15)
Other countries	6	0.74 (0.67–0.81)	0.75 (0.67–0.83)	3.0 (2.2–4.2)	0.34 (0.23–0.49)	9 (5–17)
**Population**						
AMI	7	0.73 (0.66–0.80)	0.78 (0.71–0.85)	3.3 (2.4–4.7)	0.34 (0.25–0.46)	10 (5–17)
Other population	5	0.73 (0.64–0.81)	0.76 (0.68–0.85)	3.0 (2.2–4.1)	0.37 (0.29–0.47)	8 (5–12)
**Index test**						
NT-proBNP	8	0.75 (0.69–0.81)	0.76 (0.69–0.83)	3.1 (2.5–3.9)	0.33 (0.27–0.41)	9 (7–13)
BNP	4	0.69 (0.60–0.77)	0.80 (0.71–0.88)	3.4 (2.0–6.1)	0.39 (0.27–0.54)	9 (4–20)

AMI = acute myocardial infarction, BNP = b-type natriuretic peptide, CI = credible interval, DOR = diagnostic odds ratio, NLR = negative likelihood ratio, NT-proBNP = N-terminal pro-B-type natriuretic peptide, PLR = positive likelihood ratio, SEN = sensitivity, SPE = specificity.

### 8.5. Publication bias analysis and sensitivity analysis

Deeks’s funnel plot asymmetry test demonstrated a *P* value of .25, indicating that publication bias was not statistically significant (Figure S2, Supplemental Digital Content, http://links.lww.com/MD/I189). We performed a leave-1-out sensitivity analysis to evaluate the credibility and consistency of the results, which demonstrated that the overall results were not influenced by a single study at a time, suggesting the robustness of the pooled estimate (Figure S3, Supplemental Digital Content, http://links.lww.com/MD/I190).

## 9. Discussion

This study evaluated the sensitivity and specificity of brain natriuretic peptide for predicting CIN after CAG or PCI. The results revealed that pooled sensitivity and specificity of brain natriuretic peptide index were 0.73 (95% CI: 0.67–0.78) and 0.77 (95% CI: 0.71–0.82), respectively, and AUC was 0.80 (95% CI: 0.77–0.84). Therefore, we believe that the brain natriuretic peptide index is a promising marker for identifying patients at high risk of CIN.

CIN is an impairment of kidney function that usually occurs 2 to 3 days after exposure to contrast medium. Historically, CIN was defined as an increase in the plasma creatinine level of at least 0.5 mg/dL (44 *μ*mol/L), or at minimum a 25% increase from the baseline level after exposure to contrast material.^[16]^ The Kidney Disease Improving Global Outcome working group suggested a definition based on the following criteria occurs within 48 hours after contrast administration: absolute increase in serum creatinine ≥ 0.3 mg/dL (≥ 26.4 *μ*mol/L); relative increase in serum creatinine ≥ 50% (≥ 1.5 times baseline); or urinary volume < 0.5 mL/kg/h for ≥ 6 h.^[17]^

Since there is no definitive treatment for CIN, effective preventative strategies are key to reducing the incidence of CIN, which highlights the importance of identifying high-risk patients. The Mehran risk score was developed to stratify the risk of CIN patients and included 8 variables (hypotension, intra-aortic balloon pump, congestive heart failure, chronic kidney disease, diabetes, age > 75 years, anemia, and volume of contrast).^[10]^ Although the Mehran risk score has been validated among extensive patients in multiple studies with good discriminative power, some of the included variables (such as volume of contrast medium and IABP use) are uncertain before the procedure, which limits its clinical use. Similar limitations exist in other CIN predictive models.^[8,9]^

Recently, many studies have investigated the application of biomarkers for early prediction of CIN. Neutrophil gelatinase-associated lipocalin, kidney injury molecule-1, and cystatin C are the most extensively tested biomarkers for predicting CIN.^[18–20]^ Both BNP and NT-proBNP are established heart failure biomarkers and are recommended by international guidelines.^[21,22]^ Furthermore, brain natriuretic peptide has a prognostic value for cardiovascular morbidity and mortality in patients with CAD.^[11,12]^ Therefore, BNP and NT-proBNP are common clinical test items used before interventional procedures. A Kurtul first reported that NT-proBNP level at admission was an independent predictor of CIN development after PCI in acute coronary syndrome patents.^[23]^ Liu et al investigated the predictive value of NT-proBNP for CIN in patients undergoing PCI, similar to the Mehran risk score.^[24]^

Overall, the pathophysiological mechanisms by which contrast medium causes kidney injury include direct and indirect effects. The former refers to the direct nephrotoxic effect of the contrast medium, resulting in the apoptosis and necrosis of tubular epithelial cells. The indirect mechanisms are related to ischemic injury caused by vasomotor changes.^[16,25]^ However, the underlying mechanisms of brain natriuretic peptide in predicting CIN have not been fully elucidated. Several potential explanations have been postulated. First, brain natriuretic peptide is synthesized in the cardiomyocytes, and released into the blood when the ventricular wall is stretched because of increased pressure or volume overload. Higher brain natriuretic peptide levels are associated with a reduction in cardiac output, which may affect renal artery hemodynamics. Second, as compensatory protection to counterbalance vasoconstrictor-mitogenic-sodium retaining hormones, brain natriuretic peptide is an indicator of the renin-angiotensin-aldosterone system and sympathetic nervous system,^[26]^ which may increase renal vascular resistance and decrease renal blood flow, leading to renal ischemia and hypoxia. Third, elevated brain natriuretic peptide levels were observed in patients with chronic kidney disease, but without cardiovascular abnormalities.^[27]^ In addition to the decrease in the extraction rate from blood, elevated brain natriuretic peptide levels may indicate decreasing functional renal mass and clearance receptor degradation.^[28,29]^ Therefore, patients with higher brain natriuretic peptide levels may be more vulnerable to the nephrotoxic effect of the contrast medium.

We performed a subgroup meta-analysis stratified by clinical presentation, and the results showed that acute myocardial infarction patients had a higher discriminatory accuracy than other patients. Indeed, there is evidence that the risk of CIN varies greatly with the clinical presentation. For example, patients with STEMI have a high risk of CIN, whereas those with CAD have a relatively lower risk.^[30,31]^ On the other hand, natriuretic peptide levels are higher in myocardial ischemia patients, even without ventricular dilation.^[32]^ Plasma BNP levels are elevated within 12 to 24 hours after acute myocardial infarction^[33]^ and are indicators of ventricular pressure and renin-angiotensin-aldosterone system activated by myocardial infarction, which play a role in CIN development. Age was another source of heterogeneity: the plasma BNP level is closely associated with age. Both BNP and NT-proBNP levels increased significantly with age.^[34,35]^ The prognostic value of BNP and NT-proBNP has been reported to be more significant in younger patients than in older patients.^[36,37]^ Therefore, BNP levels may have different diagnostic abilities for CIN in patients of different ages. Sources of heterogeneity were also found in the countries in which the study was conducted. It has been reported that racial differences exist in natriuretic peptide levels.^[38,39]^ Compared with white individuals, Chinese individuals have a lower NT-proBNP level.^[40]^ The index test was another source of heterogeneity. Currently, several commercial immunoassays are available for testing natriuretic peptide. NT-proBNP immunoassays have the same antibodies and calibrators with small systematic differences, whereas BNP immunoassays have different antibodies and calibrators with large systematic differences.^[41]^ Due to the lack of data on assays in some studies, we did not conduct further subgroup analyses. Further studies are needed to investigate the predictive value of different assays.

Our meta-analysis has several limitations. First, some studies had small sample sizes and all of them were observational designs, reducing the reliability of the results. Second, there was significant heterogeneity among the included studies. Although we conducted subgroup analysis to explore the potential factors responsible for heterogeneity, other factors such as the lack of a common CIN definition and different index text assays used among the studies may also be possible sources of heterogeneity. The strength of our study is that we did extensive searching studies to assess the performance of brain natriuretic peptide in predicting CIN in patients after CAG or PCI. In addition, we conducted meaningful subgroup analyses to investigate the value of brain natriuretic peptide in predicting CIN, which may guide clinical practices.

## 10. Conclusion

Our meta-analysis demonstrated that brain natriuretic peptide has excellent diagnostic value for the early detection of CIN in patients undergoing CAG or PCI. Further studies are required to determine the best cutoff value for brain natriuretic peptide in the future.

## Author contributions

**Data curation:** Xuefeng Wu, Zhaoyan Xu.

**Methodology:** Xuefeng Wu, Zhaoyan Xu, Jianming Li.

**Supervision:** Xili Yang.

**Writing – original draft:** Xuefeng Wu.

**Writing – review & editing:** Xuefeng Wu.

## Supplementary Material
