# Supplementary figures and images for: Brain natriuretic peptide as a biomarker for predicting contrast-induced nephropathy in patients undergoing coronary angiography/intervention: A systematic review and meta-analysis
Source: Medicine (Baltimore). 2022 Dec 30;101(52):e32432. doi: 10.1097/MD.0000000000032432 (PMC9803521; doi:10.1097/MD.0000000000032432)

## Slide 1
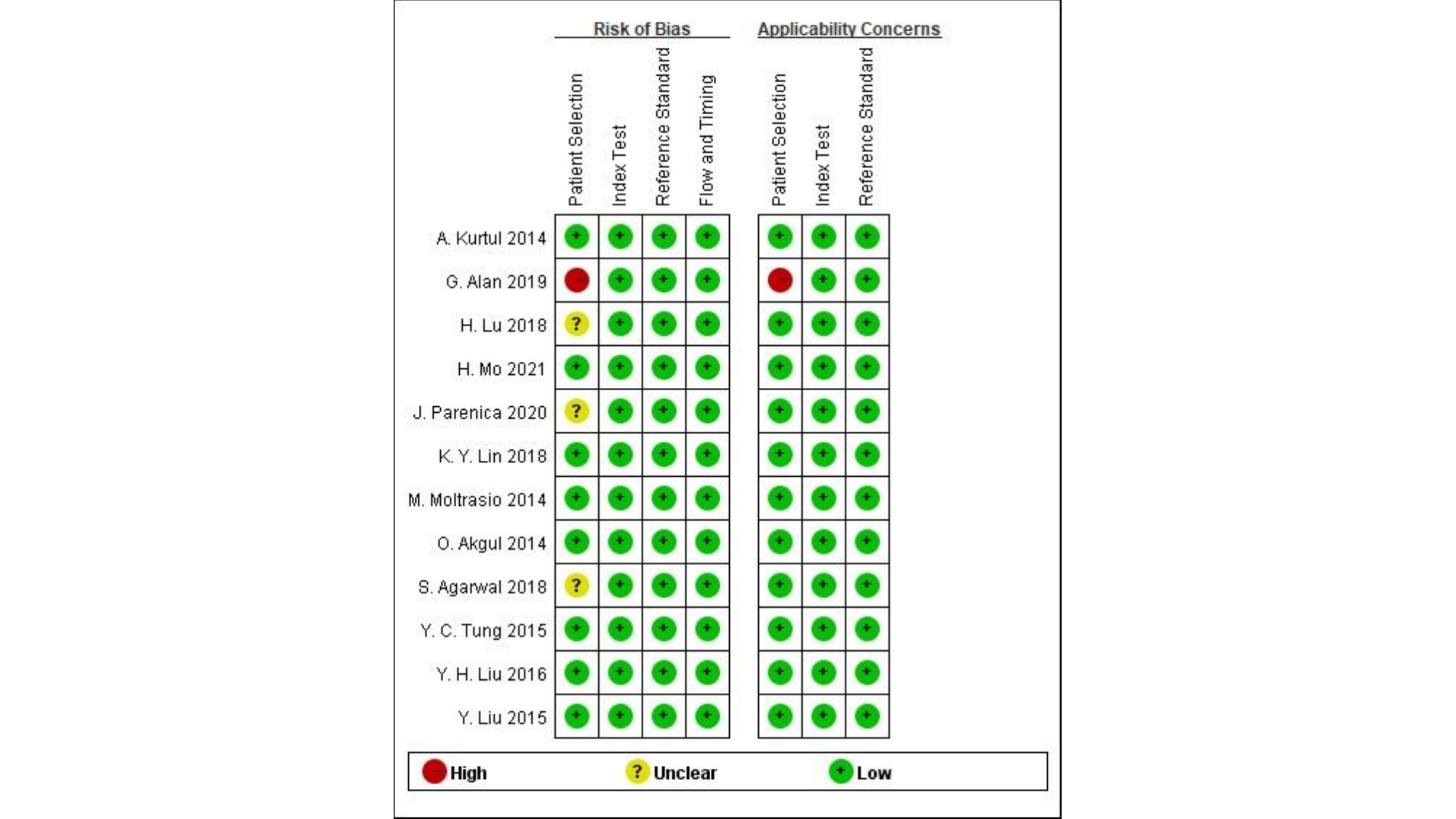

Supplement: Supplementary file 1 [file medi-101-e32432-s001.pptx]

## Slide 1
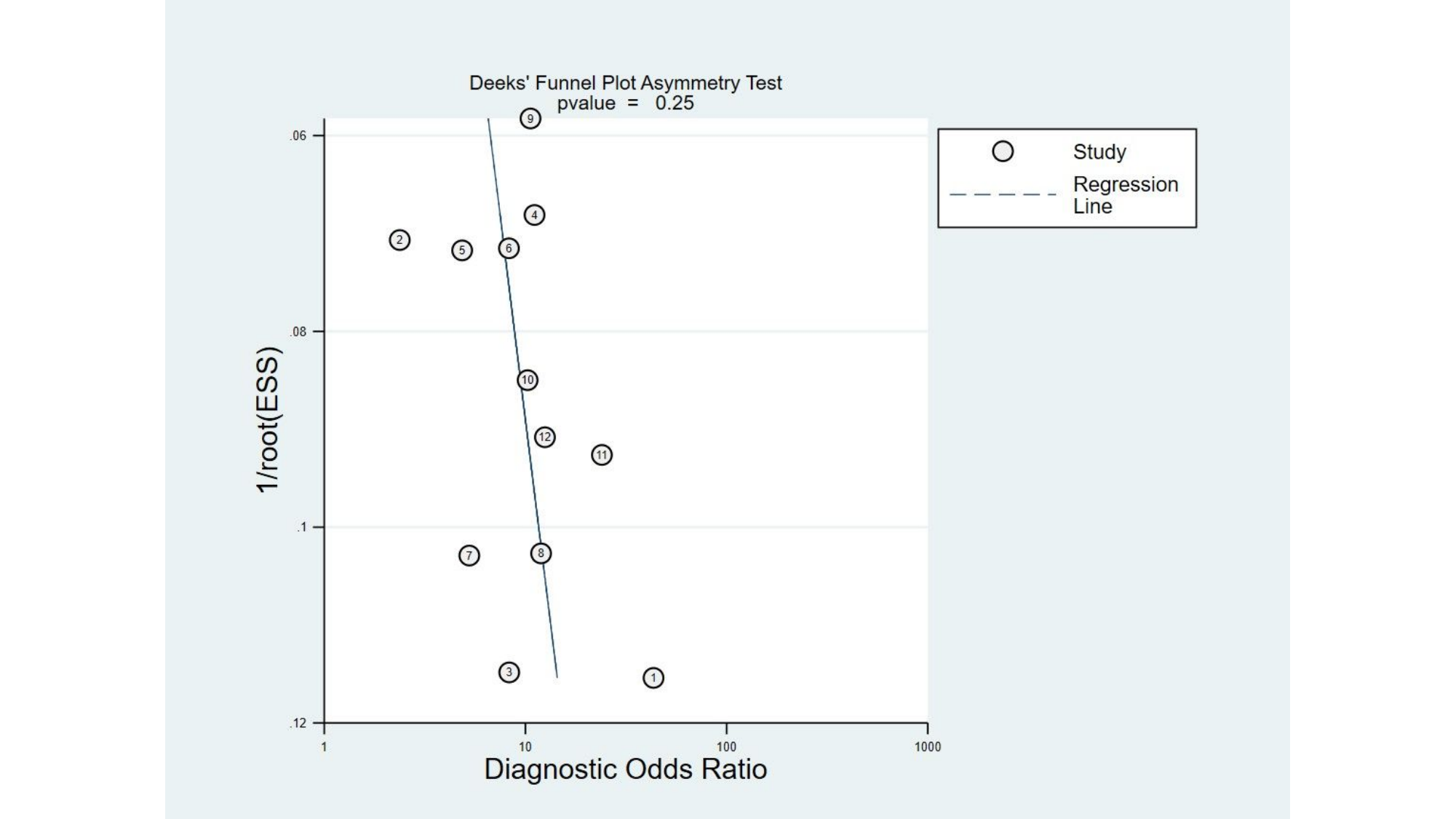

Supplement: Supplementary file 2 [file medi-101-e32432-s002.pptx]

## Slide 1
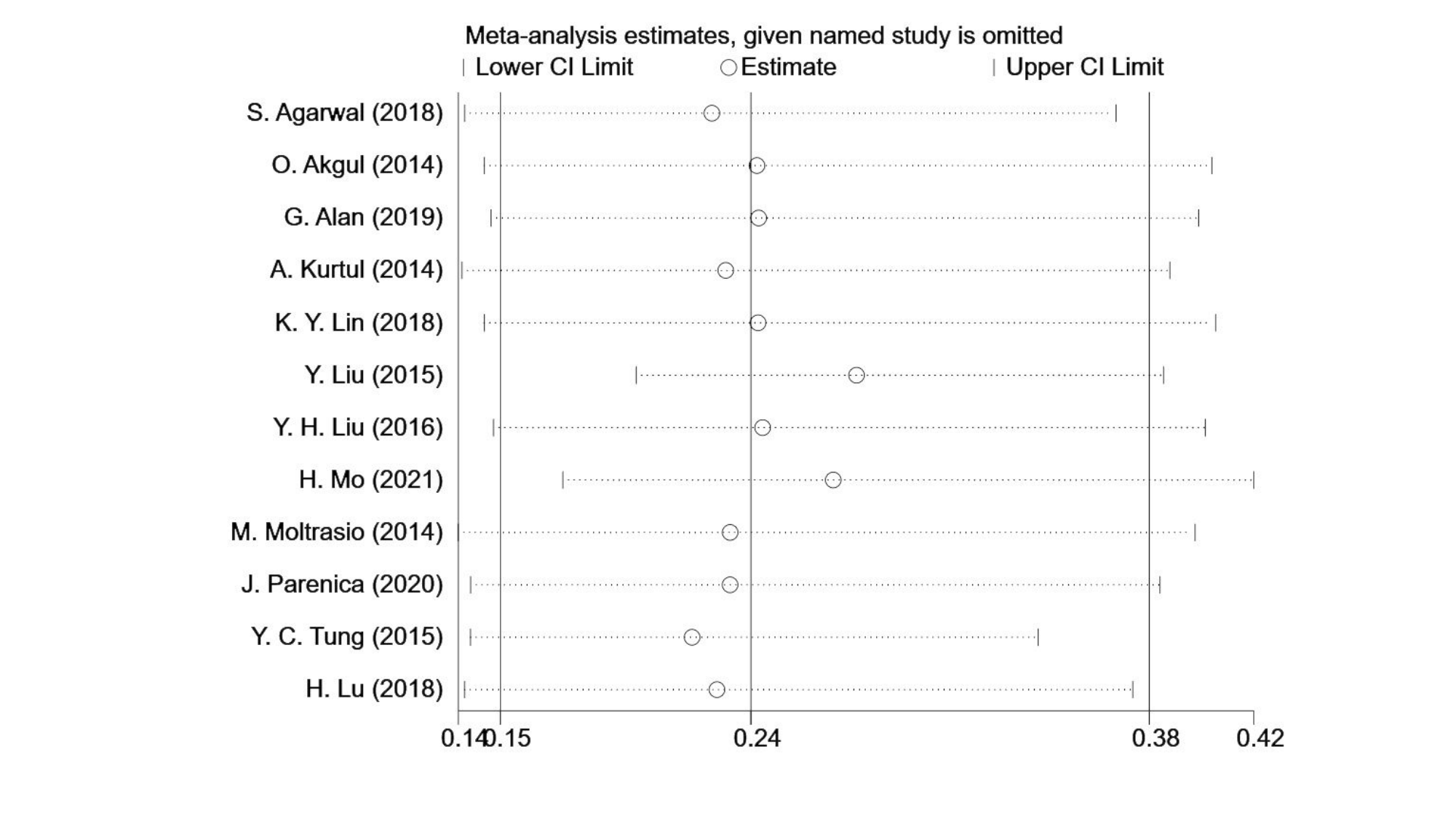

Supplement: Supplementary file 3 [file medi-101-e32432-s003.pptx]
